# *Serratia marcescens* BM1 Enhances Cadmium Stress Tolerance and Phytoremediation Potential of Soybean Through Modulation of Osmolytes, Leaf Gas Exchange, Antioxidant Machinery, and Stress-Responsive Genes Expression

**DOI:** 10.3390/antiox9010043

**Published:** 2020-01-04

**Authors:** Mohamed A. El-Esawi, Amr Elkelish, Mona Soliman, Hosam O. Elansary, Abbu Zaid, Shabir H. Wani

**Affiliations:** 1 Botany Department, Faculty of Science, Tanta University, Tanta 31527, Egypt; 2 Botany Department, Faculty of Science, Suez Canal University, Ismailia 41522, Egypt; amr.elkelish@science.suez.edu.eg; 3 Botany and Microbiology Department, Faculty of Science, Cairo University, Giza 12613, Egypt; monahsh1@gmail.com; 4 Plant Production Department, College of Food and Agriculture Sciences, King Saud University, P.O. Box 2455, Riyadh 11451, Saudi Arabia; helansary@ksu.edu.sa; 5 Floriculture, Ornamental Horticulture, and Garden Design Department, Faculty of Agriculture (El-Shatby), Alexandria University, Alexandria 21526, Egypt; 6 Plant Physiology and Biochemistry Laboratory, Department of Botany Aligarh Muslim University, Aligarh 202002, India; zaidabbu19@gmail.com; 7 Mountain Research Centre for Field Crops, Sher-e-Kashmir University of Agricultural Sciences and Technology of Kashmir, Khudwani Anantnag 192101, India; shabirhussainwani@gmail.com

**Keywords:** *Serratia marcescens* BM1, cadmium, soybean, osmolytes, antioxidants, genes expression

## Abstract

The heavy metal contamination in plant-soil environment has increased manifold recently. In order to reduce the harmful effects of metal stress in plants, the application of beneficial soil microbes is gaining much attention. In the present research, the role of *Serratia marcescens* BM1 in enhancing cadmium (Cd) stress tolerance and phytoremediation potential of soybean plants, was investigated. Exposure of soybean plants to two Cd doses (150 and 300 µM) significantly reduced plant growth, biomass, gas exchange attributes, nutrients uptake, antioxidant capacity, and the contents of chlorophyll, total phenolics, flavonoids, soluble sugars, and proteins. Additionally, Cd induced the stress levels of Cd, proline, glycine betaine, hydrogen peroxide, malondialdehyde, antioxidant enzymes (i.e., catalase, CAT; ascorbate peroxidase, APX; superoxide dismutase, SOD; peroxidise, POD), and the expression of stress-related genes (i.e., *APX, CAT*, *Fe*-*SOD, POD*, *CHI, CHS, PHD2, VSO, NR,* and *P5CS*) in soybean leaves. On the other hand, inoculation of Cd-stressed soybean plants with *Serratia marcescens* BM1 significantly enhanced the plant growth, biomass, gas exchange attributes, nutrients uptake, antioxidant capacity, and the contents of chlorophyll, total phenolics, flavonoids, soluble sugars, and proteins. Moreover, *Serratia marcescens* BM1 inoculation reduced the levels of cadmium and oxidative stress markers, but significantly induced the activities of antioxidant enzymes and the levels of osmolytes and stress-related genes expression in Cd-stressed plants. The application of 300 µM CdCl_2_ and *Serratia marcescens* triggered the highest expression levels of stress-related genes. Overall, this study suggests that inoculation of soybean plants with *Serratia marcescens* BM1 promotes phytoremediation potential and Cd stress tolerance by modulating the photosynthetic attributes, osmolytes biosynthesis, antioxidants machinery, and the expression of stress-related genes.

## 1. Introduction

Over the last few decades, there has been a growing incidence of heavy metal (HM) circulation in plant-soil continuum owing to various natural and anthropogenic activities [1]. HMs, including cadmium (Cd), pose a serious danger to the growth of crop plants, as well as human and animal health [2,3]. Cadmium is a toxic HM with strong mobility in the soil-plant interface and biological toxicity [4]. Cadmium retards vital physio-biochemical activities in plants, which include photosynthesis, biosynthesis of chlorophyll and accessory pigments, and the uptake and assimilation of essential mineral nutrients by triggering the overproduction of reactive oxygen species (ROS), including singlet oxygen (^1^O_2_), hydrogen peroxide (H_2_O_2_), superoxide radical (O_2_•^−^), and the hydroxyl radical (OH•) [5,6]. ROS accumulation causes high toxicity in plant cells [7,8,9,10,11,12,13,14,15,16]. Cadmium evokes peroxidation of membrane lipids and modulates the expression of genes of antioxidant defense systems in plants [17]. The presence of Cd inside plants thus causes stress by inducing ionic, osmotic, and oxidative stress [17,18]. Various studies also support the active involvement of antioxidant proteins in combating Cd-induced stress. Gene expression analysis further revealed the active participation of antioxidants genes (*Mn/SOD, FeSOD, POD, CAT, APX,* and *GR*) under Cd-induced stress [19,20]. 

During the course of evolution, plants have evolved intricate strategies to cope with the Cd-induced oxidative stress by activating various signalling networks, which include optimum nutrient homeostasis, enhanced accumulation of osmolytes, ROS detoxification by enhanced activities of antioxidants, and the production of thiol-related compounds [21,22,23,24]. Plant stress physiologists are engaged in devising potential sustainable strategies that can unravel mechanisms behind Cd-stress tolerance; however, the field is still emerging. Out of the various strategies that have been adopted to reverse HM-induced stress impacts in plants, the plant growth promoting rhizobacteria (PGPR) interaction is an emerging and effective sustainable way. Various recent studies in the discipline of ecological engineering and management have strongly advocated the use of PGPRs to alleviate HM-induced oxidative stress in plants, like energy crops [25], *Solanum nigrum* [26], *Spartina maritime* [27], and *Lycopersicon esculentum* [20,28,29]. Plant growth promoting rhizobacteria are known to induce tolerance against metal stress via modulating various intrinsic or underlying mechanisms [30]. These mechanisms primarily involve exclusion, extrusion, biotransformation, methylation/demethylation, and accommodation (complex formation) of metals [20,31,32,33,34]. Plant growth promoting rhizobacteria also induce the formation of different phytohormones, such as cytokinins, auxins, and gibberellins [35,36]. In addition, the synergistic interaction between microorganisms and plant roots under various abiotic pressures positively regulates the plant performance and edaphic factors [32,37,38]. *Serratia* species are potent PGPRs that are known to induce HM stress tolerance in crop plants [39]. Studies, undertaken so far, have established the role of *Serratia marcescens* in inducing HM stress tolerance in plant species. Cristani et al. [39] studied the possible action of *Serratia marcescens* in lead, Cd, and chromium metal biosorption. Khan et al. [40] analyzed the genome of *Serratia marcescens* RSC-14 and found that this species is an efficient PGPR that can alleviate Cd stress in host plants. By employing proteomic approach, Queiroz et al. [41] indicated that *Serratia marcescens* LG1 can be successfully used for adaptation and tolerance against manganese (Mn). However, the potential of the isolate *Serratia marcescens* BM1 in conferring tolerance against Cd stress in soybean (*Glycine max* L.) has not been studied yet.

Soybean is an economically important food crop worldwide and experiences various environmental stresses, including salinity and HMs, which limit the plant growth and productivity [42,43,44]. Therefore, enhancing the stress tolerance, productivity and phytoremediation potential of this crop is of utmost importance. Considering the importance of investigating these issues, the present research intends to elucidate the various underlying mechanisms that *Serratia marcescens* BM1 triggers in response to Cd stress in soybean plants. Growth traits of soybean plants have been investigated. Additionally, various physiological, biochemical and molecular approaches have been assayed.

## 2. Material and Methods

### 2.1. Investigation of Cadmium Tolerance of Serratia marcescens BM1 Strain

*Serratia marcescens* BM1 strain used in the present study was isolated from the maize rhizospheric soils of the Egyptian Suez Canal area and proved its capability for indole acetic acid production and inorganic phosphate solubilization [45,46]. In the present research, Cd tolerance of *Serratia marcescens* BM1 strain was investigated in nutrient broth media, containing 0, 150, 300, and 450 µM CdCl_2_ (i.e., CdCl_2_ was used to provide Cd stress). Bacterial growth was estimated by measuring the optical density (OD = 600 nm) following 36, 48, 72, and 96 h of incubation at 29 °C. This experiment was conducted in four replications.

### 2.2. Inoculation and Growth of Soybean Plants 

Following culturing of *Serratia marcescens* BM1 in nutrient broth media for 4 days at 29 °C, bacterial cells were centrifuged for 4 min at 2000× *g* and then collected. Resultant pellets were re-suspended in sterilized water, and bacterial culture was then set to 10^8^ colony-forming units (CFU) mL^−1^ and used to inoculate soybean plants. Soybean cultivar Giza 35 seeds were obtained from Legumes Institute of Kafrelsheikh in Egypt, and were then sterilized using sodium hypochlorite (8%, *v/v*) for 7 min, washed with sterilized water several times, and left to grow on a wet filter paper for 6 days at 24 °C. The 6-day old plants were then inoculated with *Serratia marcescens* BM1 suspension for 25 min and transferred into hydroponic plastic pots, containing Hoagland plant nutrient solution. Control plants were kept in fresh nutrient broth for 25 min. Experimental treatments were performed as follows: (i) control plants without CdCl_2_ and bacterial inoculation (T1); (ii) plants inoculated with *Serratia marcescens* BM1 alone (T2); (iii) plants treated with 150 µM CdCl_2_ alone (T3); (iv) plants treated with 150 µM CdCl_2_ and *Serratia marcescens* BM1 (T4); (v) plants treated with 300 µM CdCl_2_ alone (T5); and, (vi) plants treated with 300 µM CdCl_2_ and *Serratia marcescens* BM1 (T6). Soybean plants were irrigated with a Hoagland nutrient solution supplemented with 0, 150, and 300 µM CdCl_2_ three times a week. The pots were left in a completely randomized block design in growth chambers of a temperature of 27/19 °C (day/night) and a humidity of 76%. After 7 weeks, plants were collected for the subsequent experimental analyses.

### 2.3. Morphological Parameters of Plant Root and Shoot 

Root and shoot lengths were determined using a measuring tape. Separated roots and shoots were washed with deionized water and weighed to measure their fresh weight. Separated roots and shoots were then oven-dried at 72 °C for 50 h to estimate their dry weights.

### 2.4. Measurement of Phosphorus, Nitrogen, and Cadmium Uptake

Oven-dried leaf samples were ground into a fine powder and then digested in H_2_SO_4_ at 190 °C for 5 h. H_2_O_2_ was then added to the samples and left for 60 min. Digested samples were filtered and then diluted with sterile distilled H_2_O. Nitrogen concentration was calculated following Kjeldahl methodology as mentioned by Bremner [47]. Phosphorus concentration was calculated according to the protocol of Murphy and Riley [48]. To determine Cd content, dried leaf samples were grinded to fine powder and then digested in a mixture of HNO_3_: HClO_4_ (3:1, *v/v*) at 120 °C. Cadmium was then quantified using an atomic absorption spectrometer (AA6300C, Shimadzu, Kyoto, Japan). 

### 2.5. Measurements of Chlorophyll Content, Leaf Relative Water Content, and Gas-Exchange Attributes

Total leaf chlorophyll content was determined by homogenizing 0.2 g fresh leaf samples in 50 mL of 80% acetone, followed by centrifugation at 14,000× *g* for 7 min and the absorbance was then spectrophotometrically recorded at 662 and 645 nm as reported by Lichtenthaler [49]. Net photosynthesis rate (*P_n_*), transpiration rate (*E*), and stomatal conductance (*g_s_*) were measured on expanded leaves of similar developmental stages with a portable gas-exchange system (LI-6400, LI-COR Inc., Lincoln, NE, USA) between 9:30 and 10:30 am, according to the methodology of Holá et al. [50]. Leaf relative water content (RWC) was determined as previously explained by El-Esawi and Alayafi [12].

### 2.6. Measurement of Total Soluble Sugars, Soluble Protein, Proline and Glycine Betaine Levels

Leaves were ground into a fine powder and homogenized in 100 mM Tris buffer (pH 8.0), followed by centrifugation at at 14,000× *g* for 14 min. Using the protocol of Dey [51], total soluble sugar content was determined by recording the absorbance at 485 nm. The Bradford method [52] was used to estimate the total protein content.

The protocol of Bates et al. [53] was used to estimate proline content. Leaf samples were digested in 5% (*w/v*) sulfosalicylic acid, and centrifuged at 10,000× *g* for 7.0 min. Supernatant was diluted with sterile distilled water and then mixed with 2% ninhydrin, followed by heating up at 96 °C for 30 min, then cooling. Toluene was then added to the mixture, and the absorbance of the upper aqueous phase formed was recorded at 520 nm. Following the protocol of Grieve and Grattan [54], glycine betaine content was estimated by extracting dry leafy samples in hot distilled water at 72 °C. To the extract formed, 2 N HCl and potassium tri-iodide solution were added, mixed and cooled on ice for 2 h. Cold 1,2-dichloromethane and distilled water were then added to the mixture where two layers were formed. Organic layer absorbance was recorded at 365 nm.

### 2.7. Determination of Total Flavonoids and Phenols Contents

The protocol of Zhishen et al. [55] was used to estimate the total flavonoid content by homogenizing oven-dried leaf powder (1.0 g) in distilled water (100 mL), followed by filtration and mixing with a solution composed of distilled H_2_O, AlCl_3_, and NaNO_2_. Few drops of NaOH was then added to the mixed solution, which was then diluted with distilled water. The mixture absorbance was recorded at 510 nm. Catechin calibration curve was used.

Total phenolic content was measured by extracting leaf samples (2.0 g) in methanol solution (10 mL, 80%), followed by agitation at 72 °C for 18 min. Methanolic extract (2.0 mL) was diluted in 10 mL distilled H_2_O comprising 1 N Folin–Ciocalteau reagent (500 μL), and then incubated at 30 °C. The mixture absorbance was recorded at 725 nm [56] using gallic acid as a standard.

### 2.8. Estimation of Hydrogen Peroxide and Malondialdehyde Levels

Following the protocol of Velikova et al. [57], hydrogen peroxide (H_2_O_2_) content was measured by extracting leaf samples in 0.1% trichloroacetic acid (TCA), followed by centrifugation at 12,000× *g* for 15 min. Potassium phosphate buffer (10 mM, pH 7.0) and potassium iodide (1 M) were added and well-mixed with the supernatant. The mixture absorbance was then read at 390 nm and H_2_O_2_ content was estimated following H_2_O_2_ standard curve. Using the methodology of Heath and Packer [58], malondialdehyde (MDA) level was determined by homogenizing leaf samples in 0.1% TCA followed by centrifugation at 14,000× *g* for 6 min. Thiobarbituric acid (0.5%) and 20% TCA were added to the supernatant. The mixture was then heated up at 96°C for 25 min, followed by cooling and centrifugation at 9,000× *g* for 12 min. The supernatant absorbance was recorded at 532 and 660 nm.

### 2.9. Determination of Leaf Antioxidant Capacity 

Leaf antioxidant capacities were estimated by β-carotene-linoleic acid and 2,2′-diphenypicrylhydrazyl (DPPH) assays [59,60]. Leaf samples (2.0 g) were extracted in methanol solution (10 mL, 80%), and then agitated at 72°C for 18 min. For DPPH assay, 50 μL of methanolic extract (1 mg mL^−1^) was added to 5 mL of methanolic DPPH solution (0.004%), and then mixed and left in darkness for 25 min. The mixture absorbance was read at 517 nm. For β-carotene-linoleic acid assay, 50 μL of methanolic extract was transferred to β-carotene mixture, and then mixed and incubated for 46 h. The absorbance was read at 470 nm. The results were expressed as IC_50_ in mg mL^−1^. 

### 2.10. Estimation of Antioxidant Enzymes

Leaf samples were extracted in Tris-HCl (100 mM, pH 7.5) and then mixed with Dithiothreitol (5 mM), PVP-40 (1.5%), MgCl_2_ (10 mM), EDTA (1 mM), magnesium acetate (5 mM), phenylmethanesulfonyl fluoride (1 mM), and aproptinin (1 μg mL^−1^). The mixture was filtered and then centrifuged at 14,000× *g* for 8 min. The supernatants were utilized for estimating antioxidant enzymes activities. The protocol of Nakano and Asada [61] was used to estimate ascorbate peroxidase (APX) activity by extracting leaf samples in 2 mM AsA, followed by recording the absorbance at 265 nm. Moreover, the methodology of Aebi [62] was used to estimate catalase (CAT) activity, and the absorbance was read at 240 nm. Following the nitroblue tetrazolium photo-reduction method [63], superoxide dismutase (SOD) activity was measured, and the absorbance was read at 540 nm. Using the methodology of Putter and Becker [64], peroxidase (POD) activity was determined by recording the oxidized guaiacol production rate at 436 nm. Enzymes activities were expressed in enzyme unit per milligramme protein (EU mg^−l^ protein).

### 2.11. Expression Analysis of Stress-Related Genes 

Quantitative real-time PCR (RT-qPCR) assay was applied to investigate the expression levels of 10 stress-related genes, including *APX, CAT, Fe-SOD, POD*, *CHI* (encoding chalcone isomerase in flavonoids biosynthesis pathways; [65])*, CHS* (encoding chalcone synthase in flavonoids biosynthesis pathways)*, PHD2* (plant-homeo-domain gene of DNA binding ability and stress tolerance induction; [66])*, VSP* (encoding vegetative storage defense protein; [67]), *NR* (encoding nitrate reductase; [67]) and *P5CS* (encoding pyrroline-5 carboxylate synthetase; [67]). Total RNA was extracted from leaf tissue samples using RNeasy Plant Mini kits (Qiagen, Manchester, UK). Contaminating DNA was then removed and first-strand cDNAs were prepared using Reverse Transcription kits (Qiagen, Manchester, UK). RT-qPCR analysis was conducted as reported in the protocol of QuantiTect SYBR Green PCR kit (Qiagen, Manchester, UK). Reaction volume and PCR amplification conditions were adjusted as mentioned by El-Esawi et al. [43]. Gene-specific primers of Sirhindi et al. [68], Vaishnav et al. [67], El-Esawi et al. [69], and Kim et al. [70] (Table S1) were used for genes amplification. A housekeeping gene, *actin*, was used. The method of Livak and Schmittgen [71] was utilized to estimate relative expression levels.

### 2.12. Statistical Analysis

The experimental data are means ± standard errors (SEs) (*n* = 4). One-way analysis of variance (ANOVA) and Duncan’s multiple range test were carried out using SPSS version 16 (Chicago, IL, USA). Values at *p* ≤ 0.05 differ significantly. 

## 3. Results and Discussion

### 3.1. Cadmium Tolerance of Serratia marcescens BM1 

**Figure 1:**
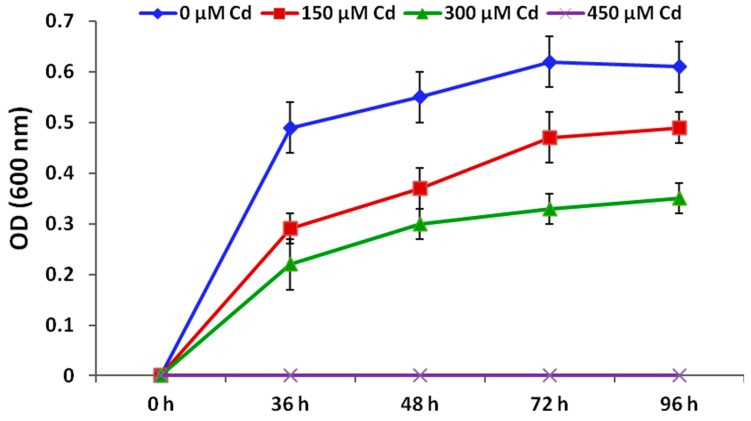
Growth curve of *Serratia marcescens* BM1 in nutrient broth media supplemented with 0, 150, 300, and 450 µM CdCl_2_ after 0, 36, 48, 72, and 96 h of incubation at 29 °C.

*Serratia marcescens* BM1 strain grew in nutrient broth medium having 0, 150 and 300 µM CdCl_2_ at different incubation time intervals with OD = 0.49–0.61, OD = 0.29–0.49 and OD = 0.22–0.35, respectively (Figure 1). However, the bacterium could not grow in the medium containing 450 µM CdCl_2_ at all the incubation time intervals (OD = 0). These findings reveal that BM1 was able to grow and tolerate Cd stress up to 300 µM CdCl_2_, indicating that this bacterium could be exploited in the phytoremediation and Cd stress tolerance studies.

### 3.2. Serratia marcescens BM1 Enhances Growth and Biomass of Soybean Plants Under Cadmium Stress

Soybean plants exhibited a significant decrease in growth and biomass traits under Cd stress at both doses (150 and 300 µM) with respect to control plants (Table 1). Cadmium stress at T3 (150 µM CdCl_2_) and T5 (300 µM CdCl_2_) treatments decreased root length by 27.56%, and 47.43% and shoot length by 30.95%, and 50.79%, respectively, with respect to the control plants. Inoculating Cd-stressed soybean with *Serratia marcescens* BM1 enhanced the length of root by 14.20% at T4 (150 µM CdCl_2_ + *Serratia marcescens* BM1) and 17.10% at T6 (300 µM CdCl_2_ + *Serratia marcescens* BM1) treatments, as compared to T3, and T5, respectively (Table 1). A similar trend also follows in case of shoot length, by which a significant increase of 18.40% at T4 and 27.40% at T6 was noticed with respect to T3, and T5, respectively (Table 1).

Root and shoot fresh weights showed a significant (*p* ≤ 0.05) decrease under Cd stress at T3 and T5 treatments compared to T1 unstressed plants (Table 1). Cadmium stress at T3 and T5 decreases the root fresh weight by 28.46% and 55.47% and shoot fresh weight by 32.24%, and 57.92%, respectively, with respect to unstressed T1 plants. On the other hand, *Serratia marcescens* BM1 application to Cd-stressed soybean plants enhanced the root fresh weight by 20.41% at T4 and 26.23% at T6 treatments with respect to T3, and T5 treatments, respectively. A similar trend was also noticed in shoot fresh weight, in which a significant increase of 13.71% at T4 and 19.48% at T6, was observed with respect to T3, and T5 treatments, respectively (Table 1).

In case of dry weights of root and shoot, Cd induces a significant (*p* ≤ 0.05) decrease at both T3 and T5 treatments (Table 1). Cadmium induced significant (*p* ≤ 0.05) reductions of 33.33% and 60.00% in root dry weight at T3, and T5 treatments, respectively, with respect to control plants. Cadmium stress also decreased the shoot dry weight by 43.24%, and 59.45% at T3, and T5 treatments, respectively, with respect to control plants. On the other hand, *Serratia marcescens* BM1 application to Cd-stressed plants showed a significant increase in root and shoot dry weight. An increase of 20.00% and 16.66% in root dry weight and 38.10% and 26.66% in shoot dry weight was noticed at T4 and T6 treatments in comparison to plants treated only with Cd at T3, and T5, respectively (Table 1). Furthermore, the root dry weight/shoot dry weight ratio was also higher for plants inoculated with *Serratia marcescens* BM1 in the presence or absence of cadmium. Therefore, *Serratia marcescens* BM1 effect was most profound on root development with a recorded increment in the root/shoot ratio in response to cadmium stress. This could be attributed to the modulation of antioxidant machinery and nutrients uptake in plant cells.

The aforementioned growth effects are consequences of the plant growth-promoting traits of *Serratia marcescens* BM1 strain, which promoted soybean growth and biomass under Cd stress conditions. Similar reports revealed the potential of *Serratia* spp. in enhancing plant growth and biomass under control and stress conditions. *Serratia marcescens* strain SRM significantly improved plant growth and biomass of wheat plants grown in cold temperature [72]. *Serratia marcescens* AL2-16 also improved the growth and biomass of the medicinal plant, *Achyranthes aspera* L. [73]. Furthermore, *Serratia liquefaciens* KM4 improved maize growth and biomass under normal and saline conditions [74]. *Serratia* marcescens CDP-13 promoted plant growth and alleviated salinity stress in wheat [75]. Moreover, Khan et al. [76] revealed that Cd stress significantly reduced plant growth and biomass of *Solanum nigrum* L. plants, whereas *Serratia* sp. RSC-14 inoculation alleviated Cd stress-induced toxic effects by significantly promoting plant growth and biomass. 

### 3.3. Serratia marcescens BM1 Modulates the Contents of Cadmium, Nitrogen and Phosphorous in Cadmium-Stressed Soybean Plants

Cadmium stress at T3 (150 µM CdCl_2_) and T5 (300 µM CdCl_2_) treatments significantly induced the accumulation of Cd over the control plants (Table 2). On the other hand, application of *Serratia marcescens* BM1 to Cd-stressed soybean plants significantly reduced Cd uptake by 21.05% at T4 (150 µM CdCl_2_ + *Serratia marcescens* BM1) and 17.86% at T6 (300 µM CdCl_2_ + *Serratia marcescens* BM1) treatments with respect to T3, and T5, respectively (Table 2). 

Under both doses of Cd stress at T3 and T5 treatments, soybean plants exhibited a decrease in N and P contents (Table 2). Cadmium stress at T3 and T5 decreased the N level by 18.18%, and 34.09%, respectively, with respect to unstressed T1 plants. *Serratia marcescens* BM1 application significantly enhanced N content at both Cd treatments. An increase of 12.50% and 12.07% was noticed in N content at T4 and T6 treatments with respect to T3, and T5, respectively (Table 2). Low and high doses of Cd stress also significantly decreased P content at T3 and T5 treatments with respect to unstressed T1 plants. Phosphorous contents showed a significant decline of 27.50% and 45.00% at T3, and T5, respectively, in relation to control plants. Application of *Serratia marcescens* BM1 to Cd-stressed soybean plants induced phosphorus content by 24.14% and 36.36% at T4 and T6 treatments with respect to T3, and T5, respectively (Table 2). These results indicate that *Serratia marcescens* BM1 was able to alleviate Cd stress-induced oxidative damage by enhancing nutrients uptake in soybean plants. Our results are in agreement with the findings of previous reports which indicated the potential of other *Serratia* strains in mitigating abiotic stress-induced oxidative damage through enhancing nutrients uptake in plants. For example, *Serratia marcescens* strain SRM significantly induced nutrients uptake of wheat plants grown under cold conditions [72]. *Serratia marcescens* TRS-1 also enhanced leaf and root phosphate content of tea plants [77]. Furthermore, *Serratia nematodiphila* LRE07 improved plant growth, biomass and nutrient uptake of *Solanum nigrum* L. plants under Cd stress conditions [78]. *Serratia liquefaciens* KM4 also significantly induced nutrient uptake of maize plants grown under normal and saline stress conditions [74].

### 3.4. Serratia marcescens BM1 Induces Leaf Gas Exchange Attributes, Leaf Relative Water Content and Biosynthesis of Chlorophyll, Sugars, Proteins, Osmolytes, Flavonoids, and Phenolics Under Cadmium Stress

*Serratia marcescens* BM1 application improved *Pn, E* and *gs* under stress-free and Cd-stressed conditions (Table 2). Soybean exposure to 150 µM CdCl_2_ (T3) and 300 µM CdCl_2_ (T5) doses significantly decreased *Pn, E* and *gs* values with respect to unstressed T1 plants. *Serratia marcescens* BM1 inoculation nullified the Cd-induced repressions in gas-exchange characteristics and enhanced *Pn, E* and *gs* at T4 (150 µM CdCl_2_ + *Serratia marcescens* BM1) and T6 (300 µM CdCl_2_ + *Serratia marcescens* BM1) treatments as compared to Cd stressed plants at T3, and T5, respectively (Table 2).

Cadmium stress decreased the chlorophyll content by 21.28% and 30.19% at T3, and T5 treatments, respectively, with respect to unstressed T1 plants (Table 3). *Serratia marcescens* BM1 inoculation significantly (*p* ≤ 0.05) induced the biosynthesis of chlorophyll by 5.66% at T4 and 5.67% at T6 treatments as compared to Cd stressed plants at T3, and T5, respectively. Furthermore, Cd stress significantly reduced the leaf relative water content (RWC) with respect to unstressed T1 plants (Table 3). On the other hand, *Serratia marcescens* BM1 inoculation significantly induced RWC in Cd-stressed plants as compared to untreated Cd stressed plants.

Low and high doses of Cd stress at T3 (150 µM CdCl_2_) and T5 (300 µM CdCl_2_) treatments in soybean plants showed a decrease in total soluble sugars and protein contents (Table 3). Both low and high dose of Cd at T3 and T5 caused significant (*p* ≤ 0.05) decreases of 9.95%, and 19.91%, respectively, in sugars levels with respect to T1 control plants. A similar trend also follows in these treatments in case of total protein content, whereas a decrease of 20.72% and 39.93% at T3, and T5 was noticed with respect to control plants. On the other hand, *Serratia marcescens* BM1 inoculation significantly enhanced sugars and protein contents at T4 (150 µM CdCl_2_ + *Serratia marcescens* BM1) and T6 (300 µM CdCl_2_ + *Serratia marcescens* BM1) treatments with respect to T3, and T5, respectively. In case of sugars, a significant increase of 5.53% and 9.84% was noticed at T4 and T6 treatments with respect to T3, and T5, respectively. Significant increases of 19.32% and 17.91% were also recorded in protein content at T4 and T6 with respect to T3, and T5, respectively (Table 3).

*Serratia marcescens* BM1 enhanced the biosynthesis of osmolytes, including proline and glycine betaine in control and Cd-stressed soybean plants (Table 3). Cadmium stress at T3 and T5 caused a marked increase in proline content by 2.69, and 3.98 fold, respectively, over control plants. A similar trend was also noticed in glycine betaine, whereas an increase of 1.76 and 2.56 fold at T3 and T5, respectively, was recorded over control plants. Inoculation of plants with *Serratia marcescens* BM1 at both Cd stress conditions had an additive effect on proline and glycine betaine biosynthesis and maximum values of these parameters were recorded at T6 treatments.

*Serratia marcescens* BM1 inoculation improved the levels of flavonoids and phenolics under control and Cd stress conditions (Table 4). Cadmium stress lowered flavonoid content by 29.95% at T3 (150 µM CdCl_2_) and by 59.77% at T5 (300 µM CdCl_2_) with respect to unstressed T1 plants. However, inoculating Cd-stressed soybean plants with *Serratia marcescens* BM1 induced flavonoids content by 10.69% and 41.37% at T4 (150 µM CdCl_2_ + *Serratia marcescens* BM1) and T6 (300 µM CdCl_2_ + *Serratia marcescens* BM1) treatments, with respect to T3, and T5, respectively (Table 4). The phenolic content also exhibited a significant decrease of 23.59% and 41.01% at T3, and T5 treatments, respectively, with respect to T1 control plants. *Serratia marcescens* BM1 application exhibited higher phenolic content of 9.55% and 24.76% at T4 and T6 treatments with respect to T3, and T5, respectively (Table 4).

The results of the above parameters demonstrate that *Serratia marcescens* BM1 also mitigated the negative impacts of Cd stress by modulating leaf gas exchange characteristics, photosynthesis, and osmolytes. These results are in agreement with the findings of previous studies which revealed the key roles of *Serratia* isolates in alleviating abiotic stress effects in plants by regulating photosynthesis, leaf gas exchange parameters, and osmolytes biosynthesis. For example, *Serratia nematodiphila* LRE07 enhanced photosynthetic pigments biosynthesis of *Solanum nigrum* L. plants under Cd stress conditions [78]. Khan et al. [76] revealed that *Serratia* sp. RSC-14 inoculation mitigated the negative impacts of Cd stress by significantly improving chlorophyll biosynthesis. Moreover, *Serratia liquefaciens* KM4 significantly induced leaf gas exchange attributes, photosynthesis process, and osmolytes biosynthesis of maize plants grown under saline stress conditions [74].

### 3.5. Serratia marcescens BM1 Inoculation Reduces the Contents of H_2_O_2_ and MDA Under Cadmium Stress

Cadmium stress at T3 (150 µM CdCl_2_) and T5 (300 µM CdCl_2_) treatments significantly increased the production of H_2_O_2_ over the control plants. On the other hand, the application of *Serratia marcescens* BM1 to Cd-stressed soybean plants significantly reduced H_2_O_2_ level by 15.27% at T4 (150 µM CdCl_2_ + *Serratia marcescens* BM1) and 29.41% at T6 (300 µM CdCl_2_ + *Serratia marcescens* BM1) treatments with respect to T3, and T5, respectively. MDA production exhibited significant increases at T3 and T5 treatments with respect to T1 unstressed plants. A significant decrease of 17.64% and 22.95% in MDA content was noticed in plants inoculated with *Serratia marcescens* BM1 at T4 and T6 treatments with respect to T3, and T5, respectively (Table 4), indicating that *Serratia marcescens* BM1 regulates membrane functions through scavenging ROS accumulation. These results are in agreement with the findings of the previous reports, which indicated the potential of *Serratia* isolates in mitigating the Cd stress impacts by reducing the oxidative stress markers, such as MDA, electrolyte leakage, and hydrogen peroxide. Khan et al. [76] revealed that *Serratia* sp. RSC-14 relived the toxic effects of Cd stress by reducing MDA and electrolytes production. Furthermore, *Serratia liquefaciens* KM4 significantly alleviated the salt stress-induced oxidative damage by reducing MDA and H_2_O_2_ contents [74].

### 3.6. Serratia marcescens BM1 Inoculation Modulates Antioxidant Capacity and Antioxidative Enzyme Activities Under Cadmium Stress

Cd stress decreased the DPPH values by 13.20% and 28.30% at T3 (150 µM CdCl_2_), and T5 (300 µM CdCl_2_) treatments, respectively, with respect to control plants (Table 4). Nevertheless, *Serratia marcescens* BM1 inoculation further caused a decrease in DPPH values by 8.69% and 7.89% at T4 (150 µM CdCl_2_ + *Serratia marcescens* BM1) and T6 (300 µM CdCl_2_ + *Serratia marcescens* BM1) treatments with respect to respective T3, and T5 treatments, respectively. β-carotene-linoleic acid of soybean plants also follows a same decreasing trend when grown under Cd stress conditions at T3 and T5 treatments. Inoculating soybean plants with *Serratia marcescens* BM1 further caused a significant decrease in β-carotene-linoleic acid values by 6.81% and by 8.10% at T4 and T6 with respect to T3, and T5, respectively (Table 4).

**Figure 2:**
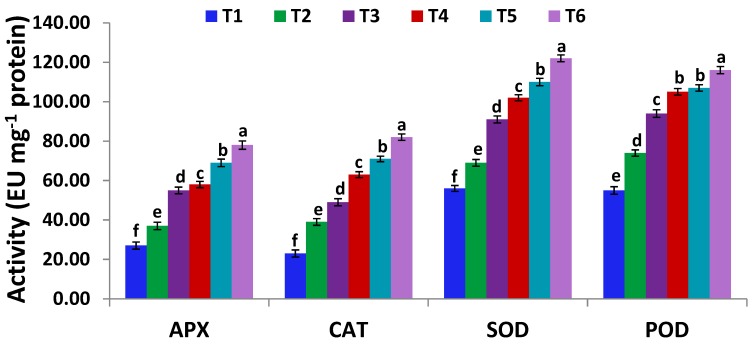
Ascorbate peroxidase (APX), catalase (CAT), superoxide dismutase (SOD), and peroxidase (POD) activities in leaves of un-inoculated and inoculated soybean plants with *Serratia marcescens* BM1 under Cd stress. Data are means ± SE (*n* = 4). Different letters (a–f) above the chart columns indicate significant differences between treatments (*p* ≤ 0.05). T1, control with no Cd and BM1 treatment; T2, plant treated with *Serratia marcescens* BM1; T3, plant treated with 150 µM CdCl_2_; T4, plant treated with 150 µM CdCl_2_ and *Serratia marcescens* BM1; T5, plant treated with 300 µM CdCl_2_; T6, plant treated with 300 µM CdCl_2_ and *Serratia marcescens* BM1.

To scavenge ROS accumulation and mitigate cellular toxicity, plants activate their defence strategies, including their non-enzymatic and enzymatic antioxidant systems, such as ascorbate, SOD, CAT, APX, and POD. In the present study, the activities of antioxidant enzymes in soybean plants, such as CAT, APX, POD, and SOD were elevated in response to Cd stress at T3 (150 µM CdCl_2_) and T5 (300 µM CdCl_2_) with respect to T1 control plants (Figure 2). Furthermore, the highest significant increase in CAT, APX, POD, and SOD activities was recorded when soybean Cd-stressed plants were inoculated with *Serratia marcescens* BM1 at T6 (300 µM CdCl_2_ + *Serratia marcescens* BM1) treatments (Figure 2). These findings are consistent with the results of the previous reports which showed the key roles of *Serratia* strains in alleviating the abiotic stress-induced oxidative damage through regulating the key antioxidant enzymes. For example, *Serratia nematodiphila* LRE07 alleviated the negative impacts of Cd stress in *Solanum nigrum* L. plants by further inducing the activities of anti-oxidative enzymes [78]. *Serratia liquefaciens* KM4 also significantly alleviated the salt stress-induced oxidative damage by upregulating various antioxidative enzymes, such as CAT, APX, POD, and SOD [74].

### 3.7. Serratia marcescens BM1 Induces the Expression Levels of Stress-Related Genes Under Cadmium Stress

**Figure 3:**
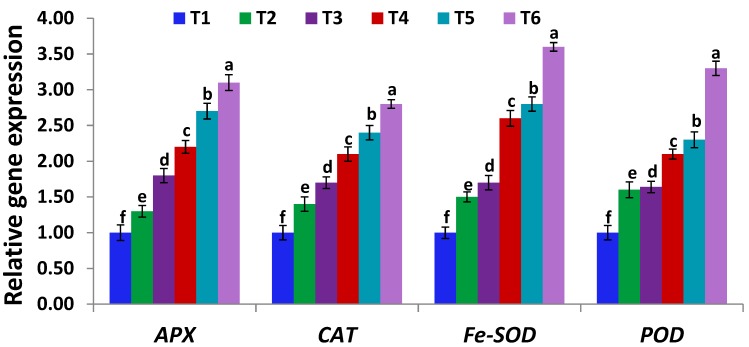
Expression levels of *APX, CAT, Fe-SOD,* and *POD* genes in leaves of un-inoculated and inoculated soybean plants with *Serratia marcescens* BM1 under Cd stress. Data are means ± SE (*n* = 4). Different letters (a–f) above the chart columns indicate significant differences between treatments (*p* ≤ 0.05). T1, control with no Cd and BM1 treatment; T2, plant treated with *Serratia marcescens* BM1; T3, plant treated with 150 µM CdCl_2_; T4, plant treated with 150 µM CdCl_2_ and *Serratia marcescens* BM1; T5, plant treated with 300 µM CdCl_2_; T6, plant treated with 300 µM CdCl_2_ and *Serratia marcescens* BM1.

**Figure 4:**
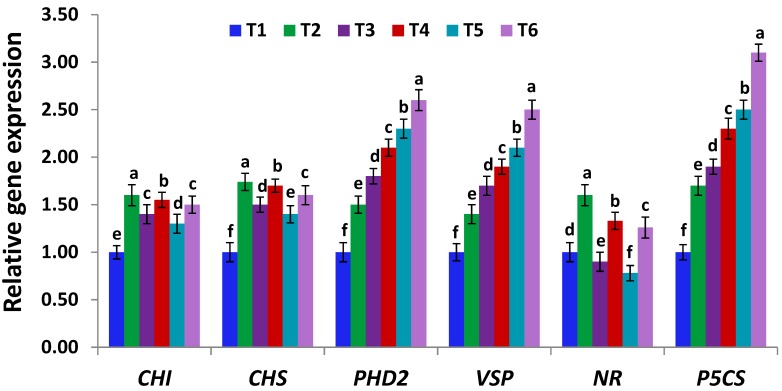
Expression levels of *CHI, CHS, PHD2, VSP, NR* and *P5CS* genes in leaves of un-inoculated and inoculated soybean plants with *Serratia marcescens* BM1 under Cd stress. Data are means ± SE (*n* = 4). Different letters (a–f) above the chart columns indicate significant differences between treatments (*p* ≤ 0.05). T1, control with no Cd and BM1 treatment; T2, plant treated with *Serratia marcescens* BM1; T3, plant treated with 150 µM CdCl_2_; T4, plant treated with 150 µM CdCl_2_ and *Serratia marcescens* BM1; T5, plant treated with 300 µM CdCl_2_; T6, plant treated with 300 µM CdCl_2_ and *Serratia marcescens* BM1.

The application of Cd stress in both doses on soybean plants exhibited a dynamic increase in the expression levels of antioxidant genes (*APX, CAT, Fe-SOD* and *POD*; Figure 3) and stress-induced genes (*CHI, CHS, PHD2, VSP,* and *P5CS*; Figure 4). The results also revealed that the expression levels of these 10 genes were further increased upon inoculating soybean plants with *Serratia marcescens* BM1, whereas, the expression levels reached to the maximum extent at T6 (300µM CdCl_2_ + *Serratia marcescens* BM1) treatments in case of *APX, CAT, Fe-SOD*, *POD*, *PHD2, VSP,* and *P5CS* genes. The expression profiling of *CHI, CHS,* and *NR* genes were induced to a maximum extent in non-stressed soybean plants, inoculated with *Serratia marcescens* BM1 (Figure 4). These results indicate the key role of *Serratia marcescens* BM1 in eliminating toxic free radicals and conferring Cd stress tolerance through upregulating the abiotic stress-related genes. Similar reports indicated the potential of *Serratia* and other PGPBs in improving abiotic stress tolerance by inducing abiotic stress-related genes [74,79].

Overall, cadmium stress-induced oxidative effects have negative impacts on gas exchange attributes and photosynthetic activity, thereby, limiting plant growth and biomass. However, the present study revealed a key role of *Serratia marcescens* BM1 in improving phytoremediation and Cd stress tolerance in soybean plants by modulating the leaf gas exchange attributes, antioxidant machinery, osmolytes, and expression of genes associated with antioxidant defence systems and stress response.

## 4. Conclusions

In the present study, Cd stress reduced the growth, biomass, photosynthetic attributes, mineral nutrients uptake, and contents of flavonoids, phenolics, soluble sugars, and proteins of soybean plants. Cadmium also increased the contents of cadmium, hydrogen peroxide, and malondialdehyde. Conversely, *Serratia marcescens* BM1 inoculation mitigated the deleterious impacts of cadmium stress by significantly inducing the plant growth, biomass, gas exchange attributes, nutrients uptake, antioxidant capacity, and the contents of chlorophyll, total phenolics, flavonoids, soluble sugars, and proteins in Cd-stressed soybean plants. *Serratia marcescens* BM1 also triggered the activities of antioxidant enzymes and the expression of stress-related genes. Considering these results, it could be concluded that *Serratia marcescens* BM1 might promote Cd stress tolerance through the modulation of photosynthesis, leaf gas exchange traits, antioxidant machinery, osmolyte biosynthesis, and the expression of stress-related genes.

## Figures and Tables

**Table 1:** Growth and biomass of un-inoculated and inoculated soybean plants with *Serratia marcescens* BM1 under Cd stress.

CdCl_2_ (µM)	BM1	Root Length (cm)	Root Fresh Weight (g Plant^−1^)	Root Dry Weight (g Plant^−1^)	Shoot Length (cm)	Shoot Fresh Weight (g Plant^−1^)	Shoot Dry Weight (g Plant^−1^)	Root DW/Shoot DW
0	−BM1 (T1)	15.6 ± 1.11b	1.37 ± 0.07b	0.15 ± 0.03b	25.2 ± 1.4b	1.83 ± 0.15b	0.37 ± 0.14b	0.41 ± 0.05b
	+BM1 (T2)	17.3 ± 1.25a	1.51 ± 0.08a	0.18 ± 0.06a	26.9 ± 1.6a	1.98 ± 0.12a	0.42 ± 0.12a	0.43 ± 0.04a
150	−BM1 (T3)	11.3 ± 1.11d	0.98 ± 0.06d	0.10 ± 0.05d	17.4± 1.3d	1.24 ± 0.11d	0.25 ± 0.15d	0.40 ± 0.05b
	+BM1 (T4)	12.9 ± 1.21c	1.18 ± 0.09c	0.12 ± 0.04c	20.6 ± 1.5c	1.41 ± 0.12c	0.29 ± 0.11c	0.42 ± 0.03a
300	−BM1 (T5)	8.2 ± 1.07f	0.61 ± 0.08f	0.06 ± 0.05f	12.4 ± 1.6f	0.77 ± 0.15f	0.16 ± 0.13f	0.37 ± 0.05d
	+BM1 (T6)	9.6 ± 1.10e	0.77 ± 0.06e	0.07 ± 0.04e	15.8 ± 1.8e	0.92 ± 0.12e	0.18 ± 0.11e	0.39 ± 0.03c

Values represent the means ± SE (*n* = 4). Different letters (a–f) next to numbers in the same column indicate significant differences between treatments (*p* ≤ 0.05). DW denotes dry weight.

**Table 2:** Minerals uptake, photosynthesis rate (*P_n_*)*,* transpiration rate (*E*) and stomatal conductance (*g_s_*) in leaves of un-inoculated and inoculated soybean plants with *Serratia marcescens* BM1 under Cd stress.

CdCl_2_ (µM)	BM1	Cd Content (mg g^−1^ DW)	N Content (mg g^−1^ DW)	P Content (mg g^−1^ DW)	*P_n_* (μmol m^2^ s^−1^)	*E* (mmol m^2^ s^−1^)	*g_s_* (mol m^2^ s^−1^)
0	−BM1 (T1)	0.01 ± 0.01e	0.88 ± 0.12b	0.40 ± 0.08b	17.03 ±1.24b	1.84 ± 0.07b	0.11 ± 0.03b
	+BM1 (T2)	0.01 ± 0.01e	0.93 ± 0.11a	0.44 ± 0.11a	18.92 ±1.15a	2.03 ± 0.05a	0.13 ± 0.02a
150	−BM1 (T3)	0.19 ± 0.09c	0.72 ± 0.09d	0.29 ± 0.09d	11.12 ±1.21d	1.49 ± 0.04d	0.06 ± 0.03d
	+BM1 (T4)	0.15 ± 0.05d	0.81 ± 0.07c	0.36 ± 0.10c	13.25 ±1.23c	1.58 ± 0.07c	0.08 ± 0.02c
300	−BM1 (T5)	0.2 8± 0.11a	0.58 ± 0.08f	0.22 ± 0.11e	8.91 ± 1.05f	1.33 ± 0.07f	0.03 ± 0.01f
	+BM1 (T6)	0.23 ± 0.10b	0.65 ± 0.07e	0.30 ± 0.12d	10.33 ±1.07e	1.42 ± 0.08e	0.05 ± 0.03e

Values represent the means ± SE (*n* = 4). Different letters (a–f) next to numbers in the same column indicate significant differences between treatments (*p* ≤ 0.05).

**Table 3:** chlorophyll content, leaf relative water content (RWC), total soluble sugars, protein, proline and glycine betaine (GB) contents in leaves of un-inoculated and inoculated soybean plants with *Serratia marcescens* BM1 under Cd stress.

CdCl_2_ (µM)	BM1	Chlorophyll (mg g^−1^ FW)	RWC (%)	Sugars (µg g^−1^ FW)	Proteins (mg g^−1^ FW)	Proline (µg g^−1^ FW)	GB (µmol g^−1^ FW)
0	−BM1 (T1)	2.02 ± 0.08b	91 ± 0.53b	2.41 ± 0.14b	1.11 ± 0.14b	20.5 ± 0.29f	2.36 ± 0.04f
	+BM1 (T2)	2.14 ± 0.07a	93 ± 0.48a	2.63 ± 0.16a	1.23 ± 0.13a	35.1 ± 0.24e	3.22 ± 0.07e
150	−BM1 (T3)	1.59 ± 0.09d	55 ± 0.51d	2.17 ± 0.14d	0.88 ± 0.10d	55.2 ± 0.41d	4.17 ± 0.06d
	+BM1 (T4)	1.68 ± 0.08c	68 ± 0.46c	2.29 ± 0.15c	1.05 ± 0.14c	77.3 ± 0.40c	5.13 ± 0.05c
300	−BM1 (T5)	1.41 ± 0.09f	42 ± 0.61e	1.93 ± 0.17e	0.67 ± 0.11f	81.7 ± 0.52b	6.06 ± 0.07b
	+BM1 (T6)	1.49 ± 0.07e	54 ± 0.45d	2.12 ± 0.13d	0.79 ± 0.14e	97.4 ± 0.55a	7.88 ± 0.06a

Values represent the means ± SE (*n* = 4). Different letters (a–f) next to numbers in the same column indicate significant differences between treatments (*p* ≤ 0.05). FW denotes the fresh weight of soybean plants.

**Table 4:** The hydrogen peroxide (H_2_O_2_) content, lipid peroxidation (MDA) level, total flavonoid content (mg catechin/g extract), total phenolic content (mg gallic acid/g extract) and antioxidant activity (DPPH and β-carotene-linoleic acid) of leaves of un-inoculated and inoculated soybean plants with *Serratia marcescens* BM1 under Cd stress.

CdCl_2_ (µM)	BM1	Total Flavonoids	Total Phenolics	H_2_O_2_ (µmol g^−1^ FW)	MDA (µmol g^−1^ FW)	DPPH (IC_50_, μg mL^−1^)	β-Carotene Linoleic Acid (IC_50_, μg mL^−1^)
0	−BM1 (T1)	7.21 ± 0.28b	17.8 ± 0.24b	0.27 ± 0.05d	20 ± 1.71e	0.53 ± 0.03a	0.51 ± 0.05a
	+BM1 (T2)	9.11 ± 0.30a	19.1 ± 0.23a	0.18 ± 0.06e	16 ± 1.66f	0.48 ± 0.05b	0.47 ± 0.04b
150	−BM1 (T3)	5.05 ± 0.22d	13.6 ± 0.22d	0.72 ± 0.04b	51 ± 2.27b	0.46 ± 0.04c	0.44 ± 0.05c
	+BM1 (T4)	5.59 ± 0.27c	14.9 ± 0.24c	0.61 ± 0.07c	42 ± 2.24d	0.42 ± 0.03d	0.41 ± 0.06d
300	−BM1 (T5)	2.9 ± 0.23f	10.5 ± 0.31e	0.85 ± 0.04a	61 ± 2.43a	0.38 ± 0.05e	0.37 ± 0.05e
	+BM1 (T6)	4.1 ± 0.26e	13.1 ± 0.29d	0.60 ± 0.05c	47 ± 2.11c	0.35 ± 0.04f	0.34 ± 0.05f

Values represent the means ± SE (*n* = 4). Different letters (a–f) next to numbers in the same column indicate significant differences between treatments (*p* ≤ 0.05).

## Supplementary Materials

The following are available online at https://www.mdpi.com/2076-3921/9/1/43/s1, Table S1: Gene-specific primers sequences used in the present study.
