# Supplementary material for: Serratia marcescens BM1 Enhances Cadmium Stress Tolerance and Phytoremediation Potential of Soybean Through Modulation of Osmolytes, Leaf Gas Exchange, Antioxidant Machinery, and Stress-Responsive Genes Expression
Source: Antioxidants (Basel). 2020 Jan 4;9(1):43. doi: 10.3390/antiox9010043 (PMC7023057; doi:10.3390/antiox9010043)
Supplement: Supplementary file 1 [file antioxidants-09-00043-s001.pdf]

antioxidants

MDPI

Table S1. Gene-specific primers sequences used in the present study.

Gene	Primer Sequence (5′-3′)	Gene Accession Number	Reference for Primer Sequence
Fe-SOD	F: ATCTTAGTTATGGTTCTCTTTGT<br>R: ATGGTGTAGAGCCTTTTCATAT	M64267	Sirhindi et al. [68]
APX	F: CGTGACGATGATTGGGAAGT<br>R: TGATAGTGATCTTTCGGACCT	NM_001354113	Sirhindi et al. [68]
CAT	F: AGCATCTCACCTGAACTTGAA<br>R: AGGTGAGAGGTTTGTGGCC	AF035252	Sirhindi et al. [68]
POD	F: TTGAAATAAAC CAAAGGAGTAGT<br>R: AATAATTATTTGAATCTCTTTAAGG	AF145349	Sirhindi et al. [68]
CHS	F: AGGCTAACAGAGGAGGGTA<br>R: CCAATTTACCGGCTTTCT	FJ770471	El-Esawi et al. [69]
CHI	F: TCCAGCGGTGGTTACTTCAC<br>R: AGTGATGGCACCGCCTTATC	AF276302	Zhou et al. [65]
P5CS	F: GGCTGCAATGCCATGGAAACTCTT<br>R: ACTTGCCTTGGGTCCTCCATACAA	AJ715851	Vaishnav et al. [67]
NR	F: TGGTTTGATGGATTCCCATT<br>R: AAATCCCATGCAAGCTCATC	NM_001251161	Vaishnav et al. [67]
VSP	F: GAAACTGATAAGGCAGGGATACA<br>R: GTTGAGAGGTGAAGGAACTACTG	M20037	Vaishnav et al. [67]
PHD2	F: AACAGGTTTTCCGGGACTTCAAGG<br>R: GCTCCTCGTCATCTTCTTCATCCA	DQ973807	Kim et al. [70]
Actin	F: CGGTGGTTCTATCTTGGCATC<br>R: GTCTTTCGCTTCAATAACCCTA	NM_001289231	Vaishnav et al. [67]
